# The significance of biomass allocation to population growth of the invasive species *Ambrosia artemisiifolia* and *Ambrosia trifida* with different densities

**DOI:** 10.1186/s12862-021-01908-4

**Published:** 2021-09-13

**Authors:** Wenxuan Zhao, Tong Liu, Yan Liu, Hanyue Wang, Ruili Wang, Qianqian Ma, Hegan Dong, Xuyi Bi

**Affiliations:** 1 College of Life Science, Shihezi University, Shihezi, 832003 Xinjiang China; 2 Xinjiang Production and Construction Corps Key Laboratory of Oasis Town and Mountain-Basin System Ecology, Shihezi, 832003 Xinjiang China

**Keywords:** Invasive mechanism, Biomass allocation, Maintenance, Fitness, Path analysis

## Abstract

**Background:**

*Ambrosia artemisiifolia* and *Ambrosia trifida* are globally distributed harmful and invasive weeds. High density clusters play an important role in their invasion. For these two species, the early settled populations are distributed at low densities, but they can rapidly achieve high population densities in a short period of time. However, their response to intraspecific competition to improve the fitness for rapid growth and maintenance of high population densities remains unclear. Therefore, to determine how these species form and maintain high population densities, individual biomass allocations patterns between different population densities (low and high), and plasticity during seedling, vegetative, breeding and mature stages were compared. In 2019, we harvested seeds at different population densities and compared them, and in 2020, we compared the number of regenerated plants across the two population densities.

**Results:**

Most biomass was invested in the stems of both species. *Ambrosia trifida* had the highest stem biomass distribution, of up to 78%, and the phenotypic plasticity of the stem was the highest. Path analysis demonstrated that at low-density, total biomass was the biggest contributor to seed production, but stem and leaf biomass was the biggest contributors to high-density populations. The number of seeds produced per plant was high in low-density populations, while the seed number per unit area was huge in high-density populations. In the second year, the number of low-density populations increased significantly. *A. artemisiifolia* and *A. trifida* accounted for 75.6% and 68.4% of the mature populations, respectively.

**Conclusions:**

High input to the stem is an important means to regulate the growth of the two species to cope with different densities. These two species can ensure reproductive success and produce appropriate seed numbers. Therefore, they can maintain a stable population over time and quickly form cluster advantages. In the management, early detection of both species and prevention of successful reproduction by chemical and mechanical means are necessary to stop cluster formation and spread.

## Background

Density is a fundamental trait of plant population distribution. Most annual invasive herbaceous plants have high population densities and usually occupy frequently disturbed habitats, forming dense monocultures by outcompeting native plants through shading [1, 2]. However, with an increase in population density, plants show strong density-dependent mortality [3, 4]. Therefore, the ability to rapidly achieve high population densities or maintain high-density advantages through individual adjustment of relevant functional traits, such as biomass and growth rate, is critical to the invasion success [5].

Flexible biomass allocation is an important mechanism for adjusting density dependence of plants. They may adjust their allocation patterns to a given environment in an economical manner [6, 7]. This allocation flexibility, in response to environmental variations, is thought to maximise the growth rate or fitness of plants to increase competition [8–10]. In dense populations, plants usually allocate more biomass to aboveground parts, maximising light assimilation and photosynthesis by increasing height [11]. A lower root to shoot ratio might confer an advantage to invasive plants by reducing intraspecific competition. In general, the larger the individual, the more seeds produced, and better dispersal [12]. Although there have been numerous studies on the effects of different densities on plant biomass allocation strategies, how biomass allocation pattern affects the maintenance or growth of the population is poorly understood [13–15].

Two invasive annual herbaceous species, *Ambrosia artemisiifolia* and *Ambrosia trifida*, have recently become troublesome plants in several regions of the world, including central and eastern Europe as well as China [16, 17]. Crop production is reduced in the invaded areas, and the large amount of pollen produced is harmful to human health. Both species are capable of forming large and dense clusters [18, 19]. Meanwhile, the seed yield per plant of *A. artemisiifolia* and *A. trifida* was large, with an average seed number of 3000–6000 and 1500–5000 per plant, respectively [16, 20]. The primary means of dispersal of *A. artemisiifolia* and *A. trifida* seeds are barochory [21]. High seed production and falling of seeds close to the mother plants (barochorie) allow the formation of dense populations [22]. The height and density of these species can produce strong shading effects that inhibit the growth of nearby species, significantly changing the biodiversity, structure and function of the invaded ecosystems [23, 24]. The formation and maintenance of such dense clusters by these species are yet to be investigated.

There are few studies on the regulation of population density by biomass allocation in *A. artemisiifolia* and *A. trifida*. Leskovsek et al. [25, 26] found that the biomass allocation of *A. artemisiifolia* is significantly affected by density, the stem partitioning coefficient increases with increasing density, whereas the root and leaf partitioning coefficient decreases. A similar change has been observed in *A. trifida*, with fewer branches and more concentrated leaves at the apex, showing the adaptive phenotypic plasticity [27, 28].

For newly settled populations of these two species, we hypothesize that biomass allocation strategies can be used to form high-density populations rapidly. This study aimed to clarify how the similarities and differences in plant biomass allocation and inter-organ interactions between the two species can effectively maintain individual growth and reproduction success, thus rapidly forming and effectively maintaining the advantages of high-density clusters in settled and mature populations.

## Results

### Variation in biomass and biomass allocation

Table 1 shows that the effects of growth stages and density, and interactions between growth stages and density on the biomass and biomass allocation traits were highly significant (P < 0.001).

**Table 1:** *F* values from two-way ANOVAs and ANCOVAs for all the biomass and allocation traits

Species	Sources	*df*	RM		SM		LM		RRM		SRM		LRM	
			*F*	*P*	*F*	*P*	*F*	*P*	*F*	*P*	*F*	*P*	*F*	*P*
*A. artemisiifolia*	S	3	318.02	< 0.001	350.72	< 0.001	345.01	< 0.001	54.89	< 0.001	28.98	< 0.001	35.42	< 0.001
	D	1	35.30	< 0.001	124.56	< 0.001	208.67	< 0.001	74.11	< 0.001	74.72	< 0.001	6.44	0.012
	C	1	3.644	0.058	2.228	0.138	1.366	0.54	1.661	0.199	0.452	0.520	0.2	0.656
	S × D	3	16.67	< 0.001	34.16	< 0.001	65.74	0.015	12.84	0.023	35.14	< 0.001	16.97	< 0.001
*A. trifida*	S	3	1205.78	< 0.001	897.82	< 0.001	512.15	< 0.001	261.96	< 0.001	135.85	< 0.001	77.49	< 0.001
	D	1	167.34	< 0.001	244.81	< 0.001	250.42	< 0.001	26.67	< 0.001	86.22	< 0.001	21.53	< 0.001
	C	1	0.002	0.967	0.019	0.892	4.77	0.063	1.421	0.235	3.699	0.056	1.355	0.223
	S × D	3	51.53	0.026	50.10	< 0.001	61.74	< 0.001	24.72	< 0.001	38.54	< 0.001	11.48	0.017

The growth stage (S), population density (D) as main effects and the sum of coverage of the main companion species (C) was considered as a covariate in two-way ANOVAs and ANCOVAs

*RM* Root biomass, *SM* stem biomass, *LM* leaf biomass, *RRM* root relative biomass; *SRM* stem relative biomass, *LRM* leaf relative biomass

**Fig. 1:**
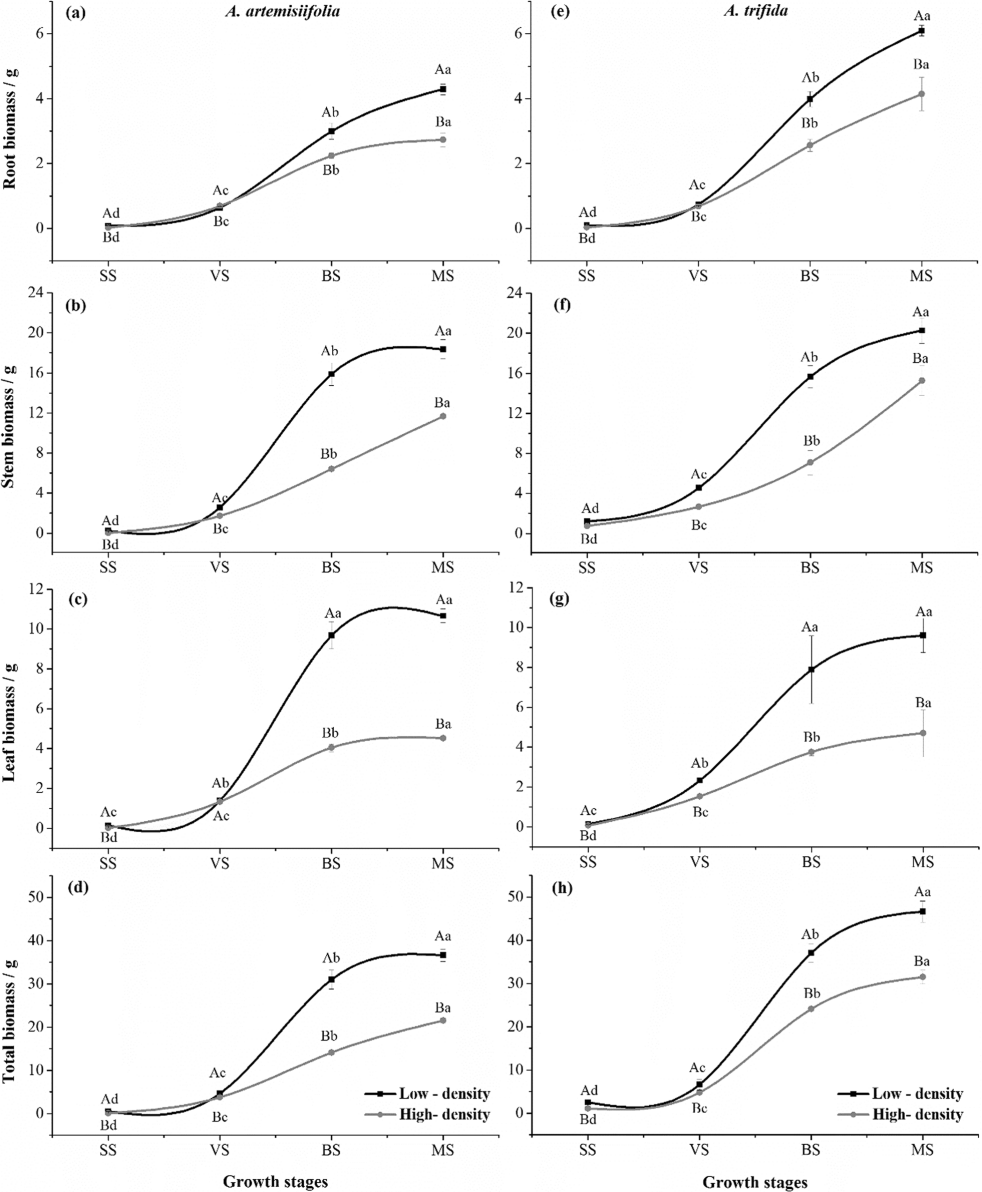
Organ and total biomass of two ragweed at different growth stages (Mean ± SE). **a**–**d** Root, stem, leaf, total biomass for *A. artemisiifolia*. **e**–**h** Root, stem, leaf, total biomass for *A. trifida*. Different lowercase letters indicate significant differences in biomass at different growth stages but the same population density (P < 0.05), while different uppercase letters indicated significant differences in at different densities but the same growth stage (P < 0.05)

Irrespective of density, the biomass of these two species increased significantly with the growth stage (Fig. 1). Plants at low-density had significantly higher root biomass (RM), stem biomass (SM), and total biomass (TM) than plants at high density for every stage. In the vegetative stage (VS), leaf biomass (LM) did not differ significantly between low- and high-density plants, but the latter had significantly higher LM at all other stages. For both species, biomass increased rapidly during VS.

**Fig. 2:**
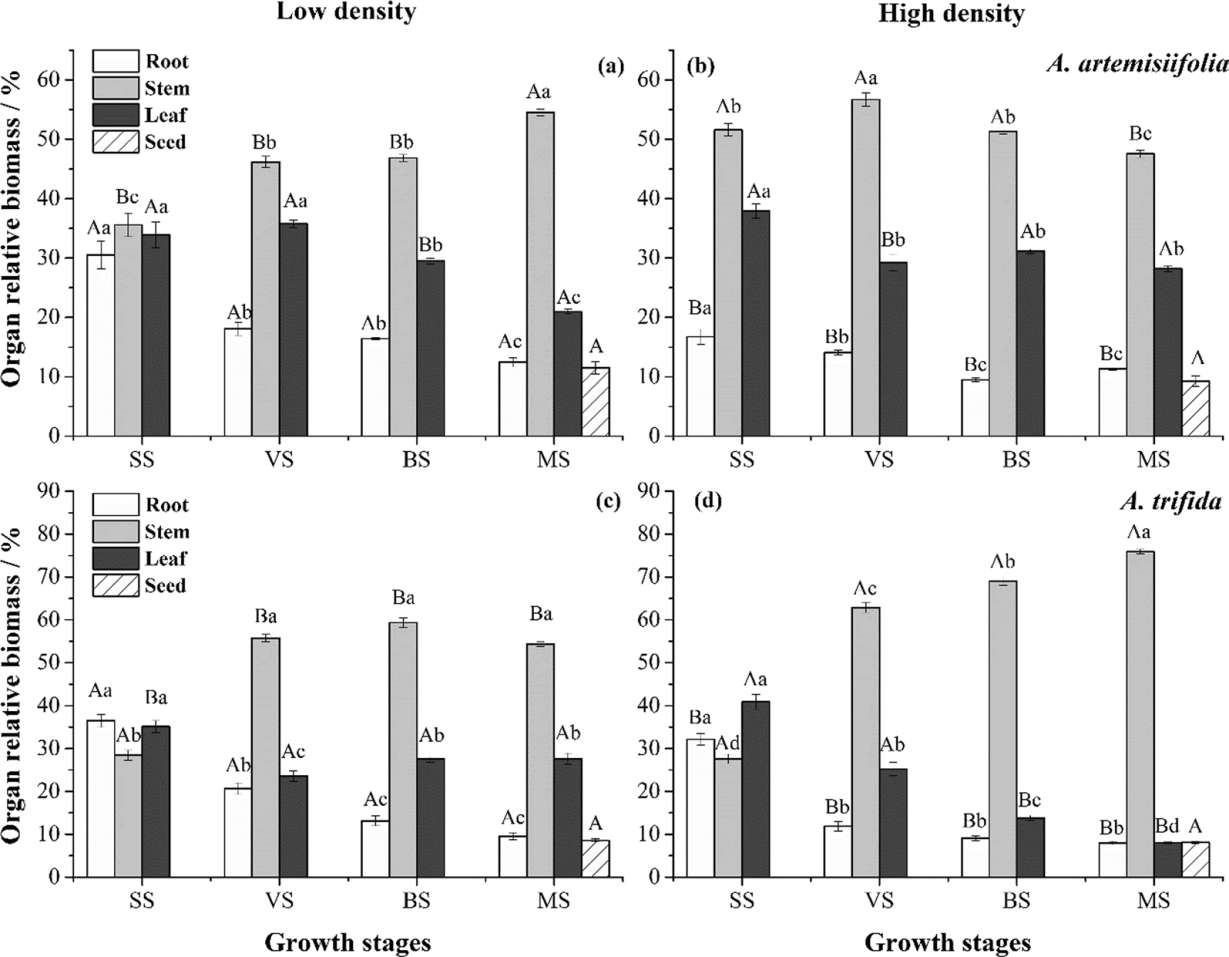
Effect of population density on biomass allocation of two ragweed organs at different growth stages (Mean ± SE). **a**, **b** *A. artemisiifolia*.; **c**, **d** *A. trifida*. Different lowercase letters indicate significant differences in relative organ biomass at the same population density across growth stages (P < 0.05), while different uppercase letters indicated significant differences in relative organ biomass across different densities during one growth stage (P < 0.05)

Relative organ biomass of *A. artemisiifolia* and *A. trifida* changed significantly as the plants grew (Fig. 2a, b). Across the season, allocation to roots and leaves declined in both species, while stem allocation was the highest throughout the growth stage and increased significantly, reaching a maximum of 55.6% at mature stages (MS) at both densities. At high-density, *A. artemisiifolia* generally had a lower allocation to roots, as well as a higher allocation to stems and leaves than at low-density. Similarly, *A. trifida* had a lower allocation to roots and higher allocation to stems at high-density than at low-density (Fig. 2c, d). Allocation to leaves was only significantly greater at high-density in the seedling stage (SS). Relative seed biomass did not differ between population densities for either species.

### Variation in phenotypic plasticity

The phenotypic plasticity index (PI) of every trait decreased significantly across growth stages, while the PI of SM showed the highest level at all stages (Table 2). For *A. artemisiifolia*, the PI of LM was the highest at the breeding stages (BS), and the PI of SM was the highest among all the characteristics at MS. However, the PI of the SM of *A. trifida* was always the largest, and the PI of the LM was also maintained at a high level.

**Table 2:** Plasticity index (PI) of growth traits in the two ragweeds species across growth stages

Traits	*A. artemisiifolia*			*A. trifida*				
	SS	VS	BS	MS	SS	VS	BS	MS
Plant height	0.74^a^	0.47^b^	0.45^b^	0.43^b^	0.77^a^	0.51^b^	0.45^b^	0.31^b^
Branch number	0^d^	0.60^a^	0.54^b^	0.48^c^	0^d^	0.58^a^	0.49^b^	0.43^c^
RM	**0.80**^**a**^	0.74^a^	0.53^c^	0.56^c^	**0.81**^**a**^	0.76^a^	0.63^c^	0.56^c^
SM	**0.98**^**a**^	**0.91**^**a**^	0.74^b^	**0.80**^**b**^	**0.98**^**a**^	**0.91**^**a**^	**0.84**^**b**^	**0.81**^**b**^
LM	**0.98**^**a**^	0.71^c^	**0.81**^**b**^	0.67^c^	**0.96**^**a**^	**0.82**^**c**^	**0.81**^**b**^	0.77^c^

*RM* Root biomass, *SM* stem biomass, *LM* leaf biomass

Different lowercase letters indicate significant differences in PI across different population densities during the same growth stage (P < 0.05)

### Contribution of plant growth traits to fitness at different population densities

**Fig. 3:**
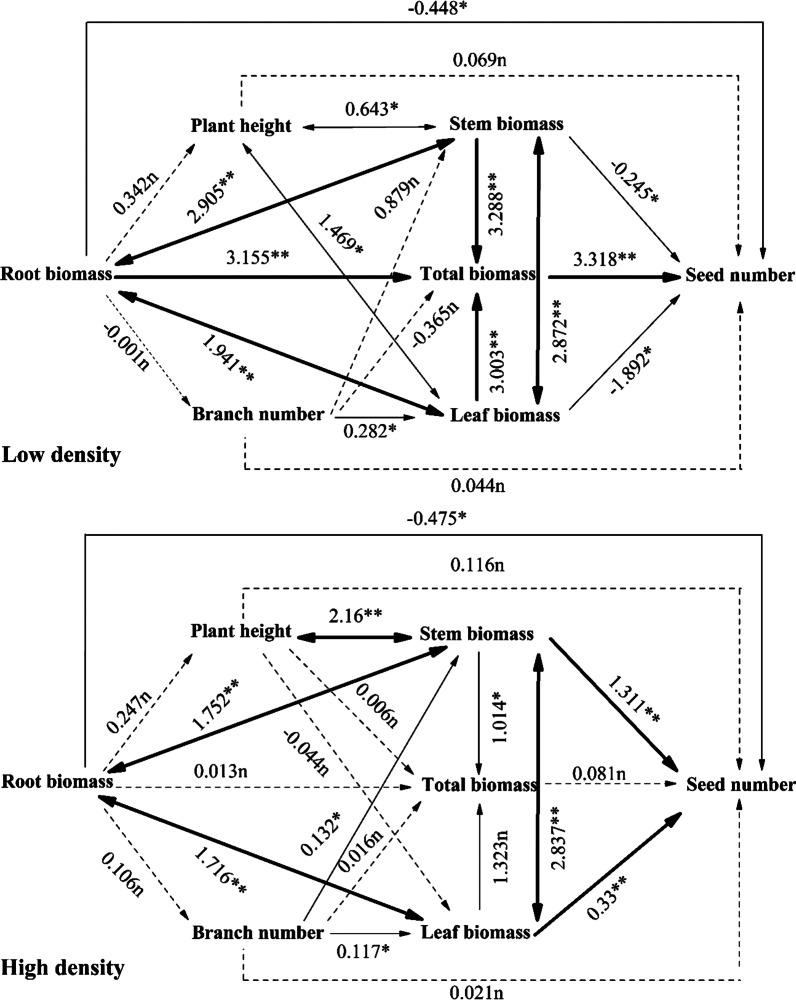
Path analysis of growth characteristics and seed number in *A. artemisiifolia* under different population densities. The dotted line indicates a path with no significant effect (n; P > 0.05), the thin line indicates a significant path (*P < 0.05), and the bold line indicates a highly significant path (**P < 0.01)

For low-density *A. artemisiifolia* (Fig. 3), the RM, SM, LM, and TM had a significant direct effect on the number of seeds, while TM had a maximum effect. The RM, SM and LM also indirectly affected the seed number through the interaction and acting on the TM. SM not only directly affected the seed number, but also indirectly affected plant height. Plant height and branch number directly affected the seed number, but not significantly. Plant height also significantly affected SM and LM and thus, indirectly affected the number of seeds. The number of branches only significantly affected the LM and, therefore, indirectly affected the number of seeds.

For high-density *A. artemisiifolia* (Fig. 3), only SM and LM directly affected seed number significantly and were positively correlated with the seed number. The RM had a significantly negative effect. The biomass of each organ had a significant effect on the TM, but the plant’s TM had no significant effect on the seed number. Plant height and branch number had a direct effect on the seed number, but it was not significant. Plant height indirectly affected the seed number by significantly affecting SM.

**Fig. 4:**
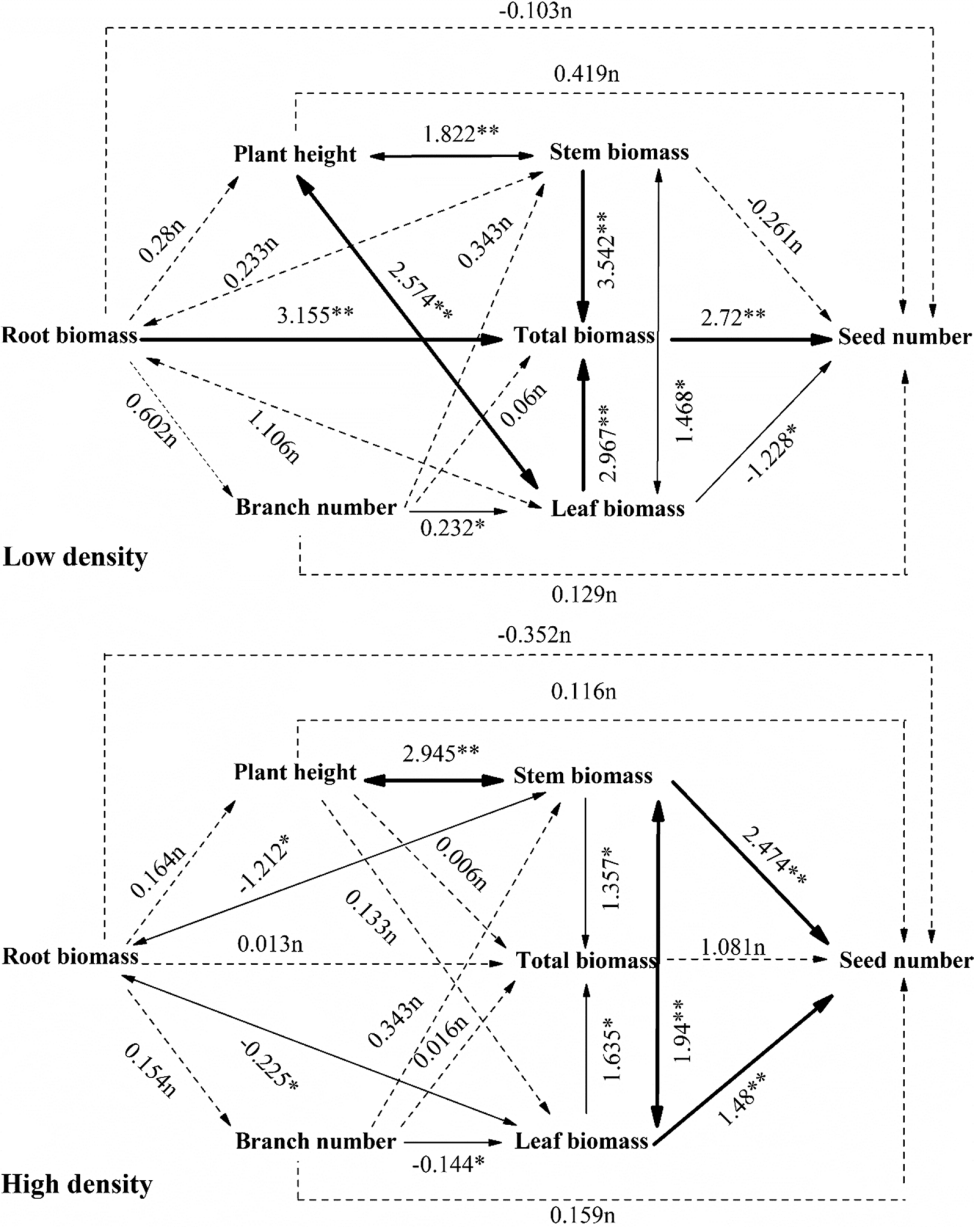
Path analysis of growth characteristics and seed number in *A. trifida* under different population densities. The dotted line indicates a path with no significant effect (n; P > 0.05), the thin line indicates a significant path (*P < 0.05), and the bold line indicates a highly significant path (**P < 0.01)

For *A. trifida* at low-density (Fig. 4), only LM and TM had significant direct effects on the seed number, LM had a negative effect, and LM had a significant positive effect. However, RM, SM, and LM affected the seed number indirectly by acting on TM. SM affected plant height, and plant height and LM had direct and indirect effects on the seed number. The number of branches only significantly affected LM and thus, indirectly affected the number of seeds.

For *A. trifida* at high-density (Fig. 4), only SM and LM had significant direct effects on seed number and were positively correlated with the seed number. The biomass of each organ had a significant effect on TM, but TM of the plant had no significant effect on the seed number. Plant height indirectly affected seed number by significantly affecting SM, and branch number indirectly affected seed number by affecting LM.

### Seed characteristics under different densities

By analysing the total number of seeds per plant and unit area of the two species (Table 3) showed that the number of seeds per plant of low-density *A. artemisiifolia* was 1.36 times higher than that of high-density plants. *A. trifida* showed similar results, producing 1.27 times more seeds per plant at low-density than at high-density. However, the total number per unit area with high-density *A. artemisiifolia* seeds was significantly higher (1.35 times) than at low-density. The total number of per unit area with high-density *A. trifida* seeds was significantly higher (1.53 times) than that of low-density.

**Table 3:** Differences in seed traits between the two ragweeds (1 m × 1 m) (Mean ± SE)

Species	Population density	Total seed numbers	
		Per plant	Per unit area
*A. artemisiifolia*	Low	5632 ± 138^a^	140,800 ± 452^b^
	High	4134 ± 106^b^	190,164 ± 433^a^
*A. trifida*	Low	3325 ± 112^a^	59,850 ± 252^b^
	High	2618 ± 86^b^	91,630 ± 363^a^

Different lowercase letters indicate significant differences between high and low densities in the same trait from the same species (P < 0.05)

### Population changes at different densities in the second year

In 2020, although the number of these two plants per unit area differed significantly across densities, the number of low-density populations increased significantly. *A. artemisiifolia* and *A. trifida* reached 75.6% and 68.4% of the mature populations, respectively (Table 4).

**Table 4:** Plant number per unit area (1 m × 1 m) across different densities during the second year (Mean ± SE)

Growth stages	*A. artemisiifolia*		*A. trifida*	
	Low-density	High-density	Low-density	High-density
SS	141 ± 5.3^Ba^	434 ± 10.5^Aa^	258 ± 4.8^Ba^	512 ± 11.5^Aa^
VS	59 ± 3.1^Bb^	101 ± 5.2^Ab^	113 ± 2.8^Bb^	435 ± 6.2^Ab^
BS	43 ± 1.3^Bc^	62 ± 2.6^Ac^	76 ± 1.4^Bc^	103 ± 1.1^Ac^
MS	34 ± 1.6^Bc^	45 ± 1.4^Ad^	26 ± 0.7^Bc^	38 ± 1.1^Ad^

Different uppercase letters indicate significant differences in plant number between high and low densities at the same growth stage (P < 0.05), while different lowercase letters indicate significant differences in plant number across different growth stages at the same density (P < 0.05)

## Discussions

### High stem input is the main growth regulator in both species regardless of their density

Annual invasive species usually occupy frequently disturbed habitats and form dense monocultures by outcompeting native plants through shading. It is important to enhance the invasive ability through interspecies competition. However, dense monocultures usually create intense intraspecific competition. The effective trade-off of biomass allocation in invasive species is very important for individual growth [29, 30]. Although high-level input to the stem was identified as the main contributor to the growth of *A. artemisiifolia* and *A. trifida*, the contributions of plant stems to individual growth and development are significantly different at different population densities.

Mature populations of *A. artemisiifolia* at high density showed strong density-dependent mortality with the advancement of the growth stage, especially in SS and VS. The rapid growth of plants in the VS, especially the increase in SM and LM, significantly increased the plant height and coverage of *A. artemisiifolia*, which grew significantly higher than the accompanying species, acquiring light resources and gaining interspecific competitive advantages (Fig. 1, Table 6). However, in the high-density population, there were few branches in the lower part of the plant owing to limited light resources, or the branches were very thin. There were few plant leaves, and with less light, most of them fell off and the plants could, therefore, only compete for light resources by growing taller. This results in plants at high density having the highest RSM of all stages, compared with that of other organs, and the SM showed strong plasticity. By increasing plant height, the growth of individual plants is maintained. *A. trifida* showed the same pattern of stem investment; however, high intraspecific competition in this species, which is taller and more expansive [31], means individuals must continuously increase their height. Therefore, RSM was higher in these species than in *A*. *artemisiifolia*.

At low density *A. artemisiifolia* and *A. trifida* were less limited by space and light resources, and the input of SM was mainly used to add more branches, resulting in more leaves and promoting the rapid growth of the individual plant. Therefore, the stem biomass allocation of the two species showed completely different patterns at high and low densities.

### Differing biomass allocation patterns at high and low densities ensure reproductive success

Annuals are generally short lived, and the rapid formation of high seed biomass ensures population survival and avoids periods of low resource supply [32]. During seed filling, stem carbohydrate reserves are depleted, and the nitrogen invested in the photosynthetic apparatus is exported after hydrolysis of proteins to amino acids, which are exported via the phloem. The gradual breakdown and export of resources invested in leaves occurs during leaf senescence and ensures remobilisation of resources previously invested in vegetative structures to developing reproductive structures [33].

Seed production of *A. artemisiifolia* is closely related to plant biomass [34]. Through path analysis, we found that low density of *A. artemisiifolia* and *A. trifida* depended on TM, while high density of *A. artemisiifolia* and *A. trifida* depended on SM and LM to ensure reproductive success. At low density, *A. artemisiifolia* are not limited by space and light resources, so plants grow fully and the individuals are larger to accumulate sufficient nutrients and energy. This is the result of multiple organs acting together from roots, stems and leaves; therefore, TM contributed the most to the seed number. However, for high density *A. artemisiifolia*, the population density reached 46 plants per m^2^ on average at the MS (Table 5) and the plant size was smaller than that of the low-density plants. To continuously increase plant height, the plant invests maximum biomass in the stem, making it more dependent on the energy stored in the stem to supply seed growth and development. Gard et al. [35] showed that both native and introduced invasive *A. artemisiifolia* tolerate artificial defoliation, which did not affect reproduction, and plants could reallocate resources in shoots even after 90% of the leaf area had been removed.

**Table 5:** The two ragweeds of different population densities during growth stages (individual/m^2^; Mean ± SE)

Growth stages	*A. artemisiifolia*		*A. trifida*	
	Low-density	High-density	Low-density	High-density
SS	235 ± 1.5	462 ± 2.8	196 ± 1.8	386 ± 3.2
VS	168 ± 1.9	396 ± 3.4	155 ± 1.3	256 ± 2.5
BS	43 ± 1.3	154 ± 2.5	45 ± 1.1	58 ± 1.7
MS	25 ± 0.6	46 ± 1.2	18 ± 0.7	35 ± 1.2

The same is true for *A. trifida*: the population density reached 35 plants per m^2^ on average at the MS (Table 5), plants are 3–4 m high, and most of the leaves are concentrated at the top, with limited photosynthesis and a high proportion of SM, which stores more energy.

### These two species can form rapidly and maintain a high population density effectively

*Ambrosia artemisiifolia* and *A. trifida* have a high number of seeds per plant and rely mainly on gravity for close dispersal [16, 20]. In our study, at low density, both species produced a high number of seeds. This enables the newly settled population to form a sizeable underground seed bank around the parent plant, laying the foundation for the rapid formation of clusters and growth [16, 36]. For high-density population plants, the plants were too dense, and the close dispersal was more obvious. The number of seeds per unit area was significantly higher than that of low-density plants (Table 3). Invaded populations for many years of *A. artemisiifolia* and *A. trifida* can produce enough seeds to replenish underground seed banks. This sets the stage for rapid cluster growth.

In the second year, although these two species experienced strong self-thinning at all stages of their growth (Table 4), the number of regenerated plants in the low-density population increased rapidly compared with the first year. At the same time, the number of plants of high-density populations did not decrease. This indicates that both species can form high-density populations rapidly and maintain them efficiently.

*Ambrosia artemisiifolia* and *A. trifida* are two plants of the same genus and different species. Although *A. trifida* is stronger in competition than *A. artemisiifolia*, *A. artemisiifolia* is distributed over a larger area compared to *A. trifida* [21]. By 2017, these two species had occupied 1322 and 311 km^2^, respectively, in the study area [37]. This difference in distribution is most likely related to the seed size and yield of the two species and their adaptation to precipitation changes. The seed size and seed weight of *A. trifida* were five times or eight times those of *A. artemisiifolia* [17]. *A. artemisiifolia* has lighter and smaller seeds; hence, *A. artemisiifolia* seeds are easier to spread in habitats with more human activity such as residential area and roadside. *A. artemisiifolia* can grow well and produce more seeds than *A. trifida* with limited water supply when the latter produces almost no seeds. When comparing the changes under simulated annual precipitation of 840 mm versus 280 mm, the seed yield per m^2^ of *A. trifida* decreased from 50,185 to 19, while that of *A. artemisiifolia* decreased from 15,579 to 530. *A. artemisiifolia* is more resistant to drought [17]. This shows that *A. artemisiifolia* has a stronger ability than *A. trifida* to tolerate drought. *A. trifida* is not well adapted to drought and it is not recorded in areas with a long summer drought. Establishment is favoured by moist environments.

Moreover, *A. artemisiifolia* is known for its ability to rapidly occupy vacant ecological niches within its invasive range; in particular, as an aggressive early colonist, it took advantage of the “priority effect” in open disturbed habitats [38] and abandoned farmland [39, 40]. This is also a reason for its larger occurrence.

Despite the distribution differences between the two species, our study found that both species maintained strong clustering distribution characteristics, with *A. artemisiifolia* up to 462 plants/m^2^ and *A. trifida* 386 plants/m^2^. The accompanying species are few and the coverage is significantly less than that of the two species (Table 2). Climate change (especially changing precipitation), degree of disturbance, characteristics of native species in the invasive habitat, and adaptability of these two ragweeds to precipitation and other environmental conditions, as well as seed size, will co-determine the distribution range of species. Maintaining high clustering ability is an important means for the two species to improve their competitiveness in the local environment. The flexible biomass allocation strategy is the basis for the formation and maintenance of the two species under high and low densities.

## Conclusions

High input to the stem is an important means to regulate the growth of *A. artemisiifolia* and *A. trifida* to cope with different densities, and *A. trifida* has more input to the stem. Both species had flexible biomass allocation patterns, which ensures high seed production and the rapid formation of high-density clusters both at the settled and mature population maintenance stages.

*Ambrosia artemisiifolia* and *A. trifida* have the ability to form monospecific and high-density stands in ruderal and grassland habitats. The present study indicates that surveys and early detection measures are necessary to determine infestations and outbreaks of both species. Moreover, preventing plant reproduction using chemical and/or mechanical means is required to stop cluster formation and rapid spread of these species. Early control is particularly important, because for larger populations, much stronger measures are necessary. Meanwhile, the suitable time of mowing is crucial as optimal management of plants must be adjusted to their phenological development to limit the quantities of released pollen and hinder their successful breeding. This is important to reduce the use of chemicals and protect the local environment.

## Methods

### Study area

The study site was located in Xinyuan County (43° 03′–43° 40′ N, 82° 28′–84° 56′E), in the hinterland of the Gongnaisi grassland in the eastern part of the Yili Valley. The average annual temperature and precipitation are 8.1 °C and 480 mm, respectively. The Yili Valley, Xinjiang, China, covers 56,400 km^2^ and contains a wide variety of habitats, including grasslands, farmlands, mountains and residential areas [41].

This site is the main distribution area of *A. artemisiifolia* and *A. trifida*. According to our previous study, these species simultaneously invaded Xinyuan County in the Yili Valley in 2010 [38], and the dominant habitat distribution of the two species varies: *A. artemisiifolia* is mainly distributed around farmland and in roadside forest belts, while *A. trifida* is mainly distributed in grassland. Since 2017, when the two species began to spread widely on a large scale, the total abundance of *A. artemisiifolia* and *A. trifida* in the Yili Valley was 57% and 39%, respectively [17].

### Study species

*Ambrosia artemisiifolia* L. is a wind-pollinated, monoecious annual herb that germinates in the spring and sets fruit in the autumn. Its height varies from 0.1 to 2.5 m tall. High phenotypic plasticity and regrowth capacity enable *A. artemisiifolia* to adapt to variable environments. Besides crop fields, it is also invading natural and semi-natural habitats and cities, and spreads along linear transport structures such as roads, railway tracks and rivers [16]. Habitat suitability and competition are likely to be the most important determinants of the number of seeds. A survey of five ragweed populations in France showed an average seed number of 2518 (± 271 SD) seeds per plant [42]. *A. artemisiifolia* is both highly noxious, due to its negative impacts on human health caused by its allergenic pollen, and an important weed of spring-sown crops [43, 44]. For example, approximately 13.5 million people suffer from common ragweed-induced allergies in Europe, causing economic costs of approximately 7.4 billion EUR annually [45]. The distributional range of this species has now expanded further into central and northern parts of the China [46].

*Ambrosia trifida* is a summer annual species 1.5–4 m in height. It is characterised by rapid growth and relatively low seed production compared to *A. artemisiifolia* [47]. *A. trifida* is also found in damp natural environments, particularly on riverbanks and floodplains as well as managed moist environments such as the banks of irrigation ditches and waterways [48]*. A. trifida* exhibits high competitiveness in various ways, such as early germination, vertically rapid growth and the formation of tall and dense canopy. Nearby species are often outcompeted from a community because water, light, nutrients, and other resources are quickly depleted. *A. trifida* is also harmful to wild and crop plants because of its competitiveness [49]. Moreover, the large amount of pollen produced by *A. trifida* is a significant human allergen and in various regions of its distribution, residents have reported allergic symptoms [31].

### Experimental design

To determine the natural adaptations of the two species to density their natural habitats (*A. artemisiifolia*: roadside forest belts, *A. trifida*: grassland) were selected as sample plots.

In April 2019, in the centre of the *A. artemisiifolia* population invasion zone, observation points with flat terrain and similar soil conditions were selected. The density of the third year of continuous intrusion was treated as high-density, while, on the periphery of the high-density population, the new dispersal area (year two) was treated as low-density (Table 5). Four quadrats (5 × 5 m) containing four plots (1 × 1 m) were each delineated in the centre of the stand. Plots were selected for uniform canopy height and density. Each quadrats was a repeated measure. The four plots within each quadrats were used for sampling at different growth stages.

*Ambrosia trifida* occurs mainly in grassland. Observation points with constant slope location and slope direction were selected (Table 5). Population density and quadrat setting were handled by the above method.

### Species collection

During the seedling stages (SS; on April 25, 2019), vegetative stages (VS; on June 15, 2019), breeding stages (BS; on August 10, 2019), and mature stages (MS; on September 25, 2019), the number and coverage of the two species in the plots were counted.

At the same time, the number and coverage of the main accompanying species were counted for every plot. The main accompanying species in the plots of *A. artemisiifolia* were *Cannabis sativa* L., *Trifolium repens* L, plants of *Artemisi*a L., and Gramineae. The main accompanying species in *A. trifida* plots were *Cannabis sativa* L., *T. repens* L, *Daucus carota* L and plants of Gramineae. *A. artemisiifolia* and *A. trifida* showed the highest coverage after the VS, significantly higher than that of the accompanying species (Table 6).

**Table 6:** Coverage of the main companion species of different population densities during growth stages (Mean ± SE)

Density	Companion species	Growth stages			
		SS	VS	MS	BS
Low	***A. artemisiifolia* L.**	2.12 ± 0.01^c^	6.53 ± 0.22^d^	16.41 ± 1.88^b^	28.49 ± 1.64^a^
	*Cannabis sativa* L.	12.83 ± 1.32^b^	15.69 ± 1.58^c^	25.10 ± 1.25^a^	33.68 ± 2.58^a^
	*Trifolium repens* L.	8.56 ± 0.97^b^	13.36 ± 0.83^b^	18.24 ± 2.41^b^	14.26 ± 1.72^b^
	*Artemisia* L.	2.78 ± 0.24^c^	5.21 ± 1.86^d^	17.54 ± 1.06^b^	11.33 ± 0.96^b^
	Gramineae	19.53 ± 2.16^a^	21.85 ± 3.14^a^	28.24 ± 1.97^a^	15.54 ± 1.60^b^
High	***A. artemisiifolia* L.**	7.36 ± 1.12^b^	21.18 ± 2.04^a^	54.93 ± 2.71^a^	52.28 ± 2.56^a^
	*Cannabis sativa* L.	8.41 ± 0.89^b^	14.32 ± 1.88^b^	18.33 ± 1.67^b^	11.62 ± 1.44^b^
	*Trifolium repens* L.	3.28 ± 0.46^c^	11.21 ± 1.60^b^	14.81 ± 2.39^b^	6.87 ± 0.98^c^
	*Artemisia* L.	9.11 ± 1.38^b^	12.56 ± 2.15^b^	10.67 ± 1.87^c^	5.34 ± 0.56^c^
	Gramineae	17.28 ± 2.03^a^	20.69 ± 2.65^a^	15.19 ± 1.72^b^	7.71 ± 0.49^c^
Low	***Ambrosia trifida* L.**	1.44 ± 0.01^d^	3.98 ± 0.13^d^	18.55 ± 0.92^b^	32.19 ± 1.21^a^
	*Cannabis sativa* L.	19.34 ± 0.62^b^	13.11 ± 0.70^c^	27.22 ± 1.04^a^	37.13 ± 0.92^a^
	*Trifolium repens* L.	10.89 ± 0.64^c^	25.08 ± 1.24^b^	21.51 ± 0.99^b^	13.22 ± 0.72^b^
	*Daucus carota* L.	1.70 ± 0.01^d^	4.53 ± 0.12^d^	7.20 ± 0.05^c^	1.40 ± 0.02^c^
	Gramineae	29.81 ± 1.01^a^	35.50 ± 3.31^a^	26.86 ± 2.40^a^	17.31 ± 1.90^b^
High	***Ambrosia trifida* L.**	9.83 ± 1.2^b^	25.09 ± 1.16^a^	66.81 ± 2.69^a^	61.31 ± 1.56^a^
	*Cannabis sativa* L.	6.33 ± 0.66^b^	4.59 ± 0.67^c^	16.28 ± 0.9^b^	13.48 ± 0.46^b^
	*Trifolium repens* L.	2.67 ± 0.73^c^	15.37 ± 1.45^b^	13.33 ± 1.45^b^	5.41 ± 0.46^c^
	*Daucus carota* L.	17.7 ± 1.21^a^	12.29 ± 1.32^b^	12.90 ± 1.07^b^	6.74 ± 0.6^c^
	Gramineae	19.44 ± 1.05^a^	19.78 ± 0.95^b^	17.31 ± 1.02^b^	8.11 ± 0.99^c^

Different lowercase letters indicate significant differences in coverage across different species during the same growth stage (P < 0.05)

To avoid the marginal effect, 30 plants of each species were randomly collected from the centre of each 1 × 1 m plot (20 cm from the boundary). Plant height was measured using a metre ruler (precision: 1 mm) before collection, and the number of branch per plant was recorded. The plants were placed in plastic bags and transported to the laboratory for further analysis. If the number of plants present was less than 30, all available individuals were collected and measured. All collected plants of *A. artemisiifolia* and *A. trifida* were identified by Wenbin Xu (Shihezi University). A voucher specimen of this material has been deposited in herbarium of Shihezi University. The deposition number of *A. artemisiifolia* is 11265 and *A. trifida* is 11272.

### Measured and calculated variables

In the laboratory, plants were cleaned and separated into roots, stems, leaves and seeds. Dry weight was determined after oven-drying for at least 48 h at 80 °C. The TM, RM, SM, and LM were measured with a 1/10,000 balance, and the relative biomass of roots (RRM), stems (SRM), and leaves (LRM) was calculated [25, 26].

The following equation was used:

1 $${\text{Relative organ biomass}} = {\text{Organ biomass }}\left( {{\text{RM}},{\text{ SM}},{\text{ LM}}} \right)/{\text{TM}}.$$

The PI for all traits was calculated as the difference between minimum and maximum means in the high- and low-density treatments, divided by the maximum mean. The PI scaled from 0 to 1, with a value closer to 1 indicates greater plasticity [50].

In MS, the number of seeds per individual in high- and low-density plots was calculated, and all seeds counted on these plants were removed. If some seeds had fallen, the number was estimated based on the locations of the seeds. For low-density plants, numbers were estimated from the number of pistillate flower clusters and subsample counts of flowers or seeds per cluster. The total seed number in plots was the sum of seed number per plant [17].

To determine the sustainability of high- and low-density populations, surviving plants were counted across growth stages during 2020 and recorded. This experiment was repeated four times.

### Statistical analysis

A general linear model (GLM) was used for the two-way ANOVA and ANCOVA (the sum of the companion species coverage) to analyse the organ biomass, total biomass and biomass allocations for differences caused by growth stages (SS, VS, BS, MS, respectively) and density (low and high density), and interactions between growth stages and density were tested. The GLM was used for the one-way ANOVA to test the difference between the biomass allocations traits of all plants due to ontogeny.

A Student’s *t*-tests were used to analyse the between-density differences in plant biomass allocation traits and seed traits as well as survival within the same growth stage. Duncan’s method was used to identify significant differences between measurements caused by the effects of ontogeny within all plants and the density (at the 0.05 level).

Through path analysis, the contribution of each growth index (plant height, branch numbers, RM, SM and LM) to fitness (seed number per individual) in both species was determined for low and high density in MS.

All data were analysed in SPSS version 19.0 and visualised using Origin version 9.0.

## Data Availability

This study was carried out with the help of Xinyuan County Government, Therefore, the datasets generated and/or analysed during the current study are not publicly available due to government requirements but are available from the corresponding author on reasonable request.

## Abbreviations

RM: Root biomass
SM: Stem biomass
LM: Leaf biomass
TM: Total biomass
RRM: Root relative biomass
SRM: Stem relative biomass
LRM: Leaf relative biomass
SS: The seedling stage
VS: The vegetative stage
BS: The breeding stage
MS: The mature stage
GLM: General linear model
PI: Phenotypic plasticity index
